# A New Method of Treatment of Diphtheria

**Published:** 1892-03-26

**Authors:** 


### THE PRACTITIONER'S MIRROR AND RETROSPECT.

[*The Editor will be glad to receive offers of co-operation and contributions from members of the profession. All letters should be addressed to* THE EDITOR, THE LODGE, PORCHESTER SQUARE, LONDON, W.]

### MEDICINE.

### A NEW METHOD OF TREATMENT OF DIPHTHERIA.

Treatment by the inhalation of vapour or gases has hitherto proved so unsatisfactory that all such methods have been rather placed in the background. Germicidal applications are more often made in the form of fluid than in vapour. But the physiological objections that may be urged against the possibility of treating pulmonary affections, such as phthisis, by inhalation, do not necessarily apply to diphtheria in which the lesion appears to be localised in the fauces and naso-pharynx or larynx. Dr. C. Smith,* of Casterton, reports excellent results from a method which, in its details, at any rate, is seemingly original.

The treatment consists in the continuous inhalation of a vapour composed of a mixture of carbolic acid, eucalyptus oil, and turpentine, until the patient is well, and at the same time support to the heart by stimulants, as well as tincture of digitalis, tincture of belladonna, and spirit ammon. aromat. In carrying out this method it is imperative that the patient be placed in bed, in order, firstly, to facilitate the inhalation; and, secondly, by the retention of the horizontal position to spare the heart as much as possible, for there is decided depression in some cases, especially if the patient be young, or the stronger mixture be used; but with proper precautions, Dr. Smith states it is not alarming. Fix a tent over the patient by arranging a sheet, or otherwise, in cases of children always, with adults generally; this should not be too large, and should be closed in on every side, except in front of his face, so that he can look about and be readily watched, and be allowed a supply of fresh air for respiration. Clothe him lightly, as the cot soon becomes warm. Mix the ingredients together in the following proportions:—Carbolic acid, 1; eucalyptus oil, 1; turpentine, 8. This is the most generally useful proportion, but greater strength combined, the author believes, with greater efficiency, may be gained by increasing the relative proportion of the two first, either to carbolic acid 1, eucalyptus oil 1, to turpentine 6, or even to turpentine 4; this is, however, decidedly more depressing, and more distinctly affects the urine—the carbolic acid, apparently, being the depressing agent. The former strength is generally sufficient, and can apparently be borne for any length of time; it is, therefore, better to use it in all cases of young children, and to return to it even in older patients after the stronger has been used for three or four days, if the weakness of the pulse or dark-clouded condition of the urine shows too much carbolic acid absorption. The judgment of the medical attendant must be exercised in this matter, but the author does not advise that the strengths 1—1—8 (weak), and 1—1—4 (strong), should be exceeded. In young children, and in all laryngeal cases, use steam continuously in the cot, either by special apparatus, or by placing a bucket at the foot, in which boiling water is renewed every half-hour day and night. In the mixture soak two cloths, linen or otherwise, about a foot square; place one close to the face, the other on the pillow near the head, on pieces of paper to prevent the soiling of the bedclothes; in adults, or children over eight or ten years of age, one or two other cloths of the same size may be soaked or hung about the cot; these must all be kept moist with the mixture, as they tend to become dry, but need not actually be dripping. Care must be taken that they do not touch the face, although very close to it, otherwise the skin will become burnt. The nurse must watch to see that they are always sufficiently near to give off a strong odour close to the patient's mouth, or several cloths must be used in the tent so that it may be filled with the vapour. If old enough to keep the mouth open he should do so voluntarily, a piece of cork or wood being placed between the back teeth to assist him, for there is considerable difficulty in maintaining the mouth open in this position. In young children it may be necessary to plug the nostrils with wool. The impact of the vapour on the diseased spot is essential to cure, and by this method this is facilitated. Sometimes the child will talk or sing, while at others he will simply sit in his cot and roar; in either case plugging of the nostrils is not necessary, but it is often of the greatest assistance, and should seldom be omitted, especially during sleep. This, of course, applies only when the deposit is chiefly on the tonsils or soft palate; when laryngeal only, it is necessary, and even if pharyngeal as well, it will generally be safe to omit, at any rate, the nostril plugs, for the cure of the laryngeal portion of the disease takes so long that the pharyngeal portion almost certainly disappears at the same time. Should there be nasal diphtheria also, the nostril plugs must be omitted, at any rate at intervals. The vapour must be applied in the manner directed, and in no other.

In some cases the heart depression is considerable, especially if the 1·4 strength be used, or the case be long continued. To combat this give from the commencement of the case brandy or other spirit in full but regulated doses, and tincture of digitalis and belladonna, with spir. ammon. aromat., at intervals of four hours, and in quantities suited to the age of the patient, also as much food as he can be persuaded to take.

From four or five to ten days are required for a cure, only the very earliest seen cases taking less than four; laryngeal ones generally take longer than pharyngeal. The effects of treatment begin to show themselves within twenty-four hours; supposing the case to have been a bad pharyngeal one, with extensive deposit, and the 1·1·4 mixture to have been used without steam. First the intervals between the patches gradually clear up and become red. Secondly, the deposit becomes altered in colour, grey and dead looking; the edges loose and well-defined, and gradually their size diminishes. There is no expectoration of the false membrane, and no coming away in pieces, it simply dissolves away and disappears, leaving the throat intensely red and sore. Coincident with the local improvement is a general one; the fever disappears, the general condition improves, appetite increases, and the patient becomes lively and cheerful.

In laryngeal cases, steam should always be used; the membrane softens down, and is expectorated in a thick creamy or ropey phlegm; this process continuing so long as any deposit remains in the trachea. In these cases, if far advanced, there is sometimes considerable difficulty in breathing at the commencement of treatment, owing to the broken-down membrane having to escape through an already narrowed passage. Great benefit will often arise in such cases from the passage down to the glottis of a small pad of lint fixed to a bent wire and dipped in tincture of iodine.

Dr. Smith draws special attention to the limitations of his method. The membrane has to be got rid of, and it is softened down ready for expectoration by this method; but should the previous duration of the disease have been such that the bronchial tubes are lined extensively with it, the amount to be thrown off is too much for the possibility of recovery, particularly if the patient be very young, he dies, drowned by the disintegrating membrane, even while under-

* *Australian Med. Journ.*, Oct. 15th, 1891. *Pract.*, Dec., 1891.

going a process of cure. Three or four days of the laryngeal variety, before the treatment is commenced, is as much as we can expect our patients to recover from; if pharyngeal, the author believes there is no amount or duration of the disease that may not be recovered from by this method. Of the seventeen cases treated by this method, Dr. Smith lost only two, both aged three years. But seven of the cases were under five years of age; five recoveries and two deaths from diphtheria in such young patients is, indeed, a most gratifying result.

In introducing his paper, Dr. Smith remarks: "In bringing under your notice a method of treating diphtheria which is new, easy of application, safe, and, with some slight reservations, infallible, I am prepared to meet with incredulity; such is the correct attitude of the scientific mind when confronted with a new fact or theory. I have, however, by observation and experiment, so thoroughly tested the truth of the one I now submit to you, that I do so with the greatest confidence, feeling sure that the result of investigation on your part will be to establish it on a wider and surer basis than before." We trust the experience of those who essay Dr. Smith's method, may obtain results as satisfactory as the originator.

